# Clinical significance of urinary L-FABP in the emergency department

**DOI:** 10.1186/s12245-019-0244-9

**Published:** 2019-08-30

**Authors:** Ginga Suzuki, Ryo Ichibayashi, Saki Yamamoto, Yoshimi Nakamichi, Masayuki Watanabe, Mitsuru Honda

**Affiliations:** Emergency and Critical Care Center, Toho University Omori Medical Center, 6-11-1 Omori-Nishi, Ota-ku, Tokyo, 143-8541 Japan

**Keywords:** Liver-type fatty acid-binding protein, L-FABP, Acute kidney injury, AKI

## Abstract

**Background:**

This study’s aim is to measure liver-type fatty acid-binding protein (L-FABP) levels in urine using a rapid semiquantitative assay kit in the emergency department and to investigate whether the onset of acute kidney injury (AKI) after hospitalization can be predicted.

**Methods:**

This was a prospective observation study. Patients transferred to the emergency and critical care center were divided into two groups: urinary L-FABP negative and positive groups. The status and severity of AKI were evaluated for the respective patients based on the Kidney Disease: Improving Global Outcome (KDIGO) classification. We compared the proportion of AKI patients in the two groups.

**Results:**

In the urine L-FABP-positive group, many patients had a significant onset of AKI (*p* < 0.001). After excluding patients who were diagnosed as AKI for creatinine level at admission, urinary L-FABP could predict the onset of AKI after admission (*p* < 0.001).

**Conclusion:**

By measuring urinary L-FABP concentration using a rapid semiquantitative assay kit, there is the possibility that the onset of AKI after admission can be predicted from immediately after a patient is transported by ambulance.

## Introduction

The diagnosis and severity of acute kidney injury (AKI) are defined based on the Risk, Injury, Failure, Loss, End-stage kidney disease; Acute Kidney Injury Network; and Kidney Disease: Improving Global Outcomes (KDIGO) classifications. All these classifications are based on serum creatinine levels and urine output. Moreover, a study reported that an increase in serum creatinine level may not be seen in 24–72 h after an invasion [1]. During that time, one needs to quickly notice any progression in the renal disorder. Meanwhile, the novel AKI biomarkers are drawing attention, and one of them is urine liver-type fatty acid-binding protein (L-FABP) [2–4].

Urinary L-FABP has been reported to enable early prediction of the onset of AKI with the conventional diagnostic criteria after open heart surgery and in sepsis [5–8]. However, no studies on urinary L-FABP in emergency and critical care centers to which critically ill patients are transported have been conducted. Moreover, assay of urinary L-FABP requires at least a few days, and during that time, quickly predicting the progression of AKI and its severity is difficult. Therefore, this study aimed to investigate whether AKI onset after admission from immediately after a patient is transported to an emergency and critical care center can be predicted by measuring the urinary L-FABP of the patient using the rapid semiquantitative assay kit.

## Materials and methods

### Study design and setting

A prospective observation study was conducted involving 176 patients who were transported to the Emergency and Critical Care Center, Toho University Omori Medical Center, between September 1, 2017, and April 12, 2019. This hospital is a tertiary medical care facility located in Tokyo, Japan. The study protocol was approved by Toho University Omori Medical Center ethics committee (approval number is M17084), and written informed consent was obtained from all the participants.

### Selection of participants

The study participants included 250 consecutive patients who were transported to the emergency and critical care center and underwent insertion of a urethral catheter. The exclusion criteria were patients below 18 years of age, patients on maintenance dialysis, patients who underwent kidney transplant, patients for whom “do not attempt resuscitation (DNAR) when hospitalized” was ordered, and patients whose in-hospital duration is < 48 h. Since at least 48 h is required to diagnose and judge the severity of AKI, based on the protocol, patients who died or were discharged within 48 h were also excluded.

### Measurement and data collection

**Fig. 1:**
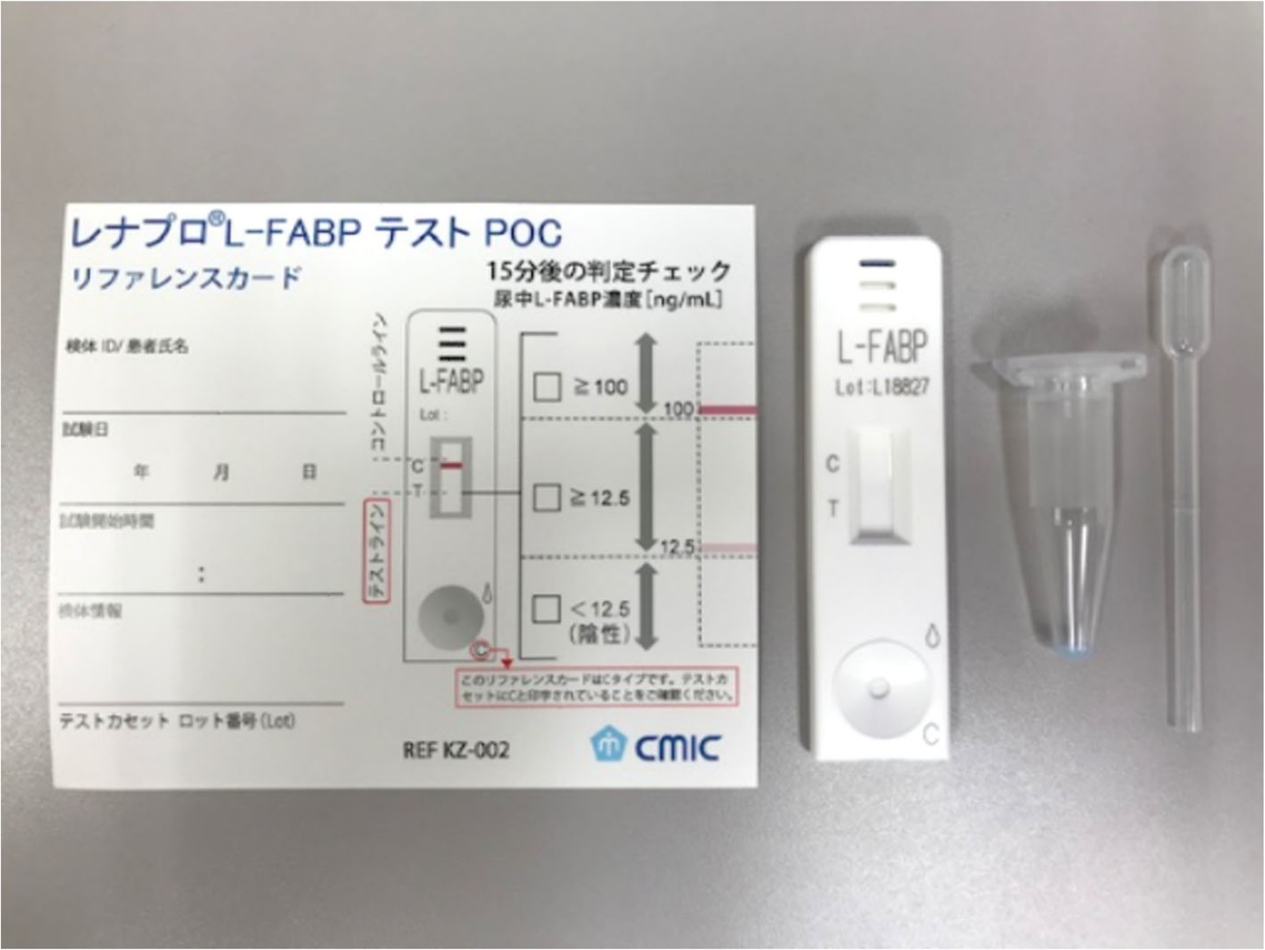
The kit contains these items. Usage is in accordance with the instruction manual

Samples were collected through insertion of a urethral catheter at the initial treatment. A rapid semiquantitative assay kit, RENAPRO^Ⓡ^ (CMIC Pharmaceutical Services Co., Ltd., Tokyo, Japan), was used to measure urinary L-FABP concentration (Fig. 1). Values < 12.5 ng/ml were considered as negative, those ≥ 12.5 ng/ml but < 100 ng/ml as weakly positive, and those ≥ 100 ng/ml as strongly positive. In this study, weakly positive and strongly positive were judged as positive. To prevent errors due to degeneration, the measurement was performed using the kit immediately after the urine sample was collected.

The following items were extracted from the medical records and evaluated: age, sex, status of diabetes, underlying disease responsible for the hospitalization, use of a contrast agent at admission, status of oral treatment with an angiotensin-converting enzyme (ACE) inhibitor or angiotensin II receptor blocker (ARB), status of oral treatment with a loop diuretic, status of oral treatment with non-steroidal anti-inflammatory drugs (NSAIDs), blood pressure and pulse rate at admission, serum creatinine level (at the hospital visit and baseline level), duration of intensive care unit (ICU) stay, use of renal replacement therapy (RRT), 28-day mortality rate, Acute Physiology and Chronic Health Evaluation (APACHE) II score, stage based on the KDIGO classification, and number of persistent AKI. Sepsis was diagnosed as the underlying diseases using the sequential organ failure assessment (SOFA) score and quick SOFA according to the Third International Consensus Definitions for Sepsis and Septic Shock (Sepsis-3) [9]. And persistent AKI was defined as stage 2 or 3 AKI. The AKI status was judged within 7 days with creatinine level and urine output criteria, and those with stage 1 or more AKI in the KDIGO classification were judged as AKI. When the baseline serum creatinine level was unknown, if the level improved quickly after admission, that level was used. If there was no improvement, the value that was calculated by the Modification of Diet in Renal Disease equation formula and GFR set at 75 ml/min/1.73m^2^ was used for the sake of convenience [10, 11].

### Outcome measures

The primary outcome was the onset of AKI, and the secondary outcomes were the duration of ICU, use of RRT, 28-day mortality, and onset of persistent AKI.

### Statistical analysis

All the data showed a non-normal distribution. The continuous variables were expressed as median and quartile values, while the nominal variables and ordinal variables were expressed as percentages. For the continuous and ordinal variables, the Mann–Whitney *U* test was used. For the nominal variables, *χ*^2^ test or Fisher’s exact test was used. In all the tests, *p* values < 0.05 were considered as statistically significant. For the above analyses, StatFlex version 6.0 for Windows (Artek K. K, Tokyo, Japan) was used.

## Results

Two hundred and fifty consecutive patients who were transported to the emergency and critical care center during between September 1, 2017, and April 12, 2018, and who underwent insertion of a urethral catheter were enrolled in the study. One patient was below 18 years of age, one was on maintenance dialysis, DNAR was ordered for 23 patients at admission, the duration of hospitalization was < 48 h in 28 patients, and 21 patients had inadequate measurements. The inadequate measurements continued for at least 3 h from the transportation of the patients to sample collection and at least 3 h passed from the measurement to the judgment. These were the cases where control of the kit could not be displayed. As a result, a total of 176 patients were included in the final analysis.

Significant differences were observed between the two groups that were divided by urinary L-FABP concentration regarding serum creatinine level, rate of use of the RRT, APACHE II score, KDIGO class, and rate of persistent AKI. No significant difference was observed in terms of age, sex, use of a contrast agent, and baseline serum creatinine level, and no difference was also observed regarding underlying disease and status of diabetes. Moreover, no difference was observed in drugs that affect kidney function (ACE inhibitors, ARBs, loop diuretics, and NSAIDs). The 28-day mortality rate also showed no significant difference (Table 1). Rate of onset of AKI (stage 1 to 3) was higher in L-FABP-positive group (41.5% vs 83.0%, *p* < 0.001). The median of creatinine at admission was already 1.5 times higher than the baseline creatinine in the positive group. So we exclude the patients who already progress to AKI at admission.

**Table 1:**
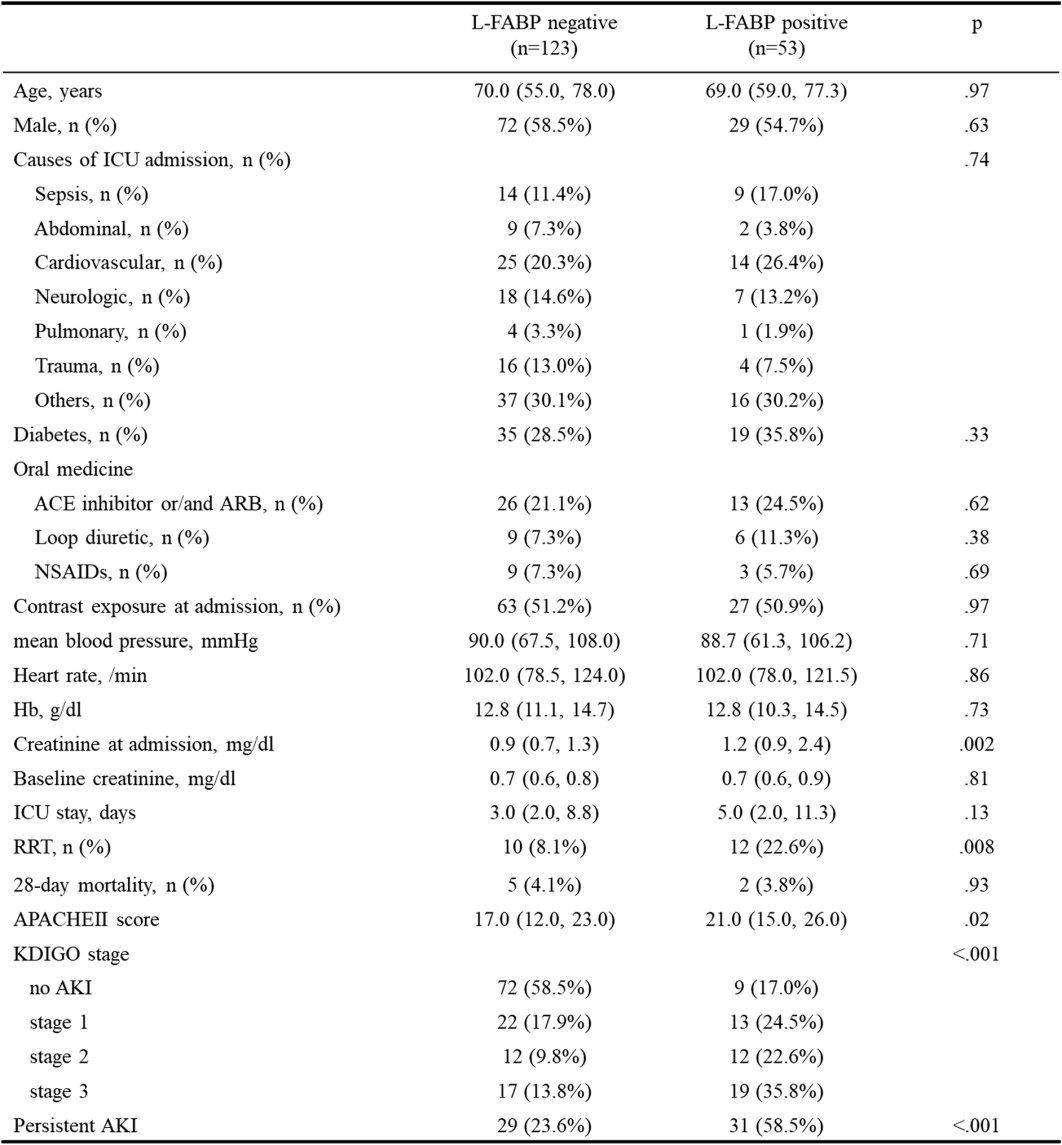
Baseline clinical characteristic

*L-FABP* liver-type fatty acid-binding protein, *ICU* intensive care unit, *ACE* angiotensin-converting enzyme, *ARB* angiotensin II receptor blocker, *NSAIDs* non-steroidal anti-inflammatory drugs, *RRT* renal replacement therapy, *APACHE* Acute Physiology and Chronic Health Evaluation, *KDIGO* Kidney Disease: Improving Global Outcomes, *AKI* acute kidney injury

**Fig. 2:**
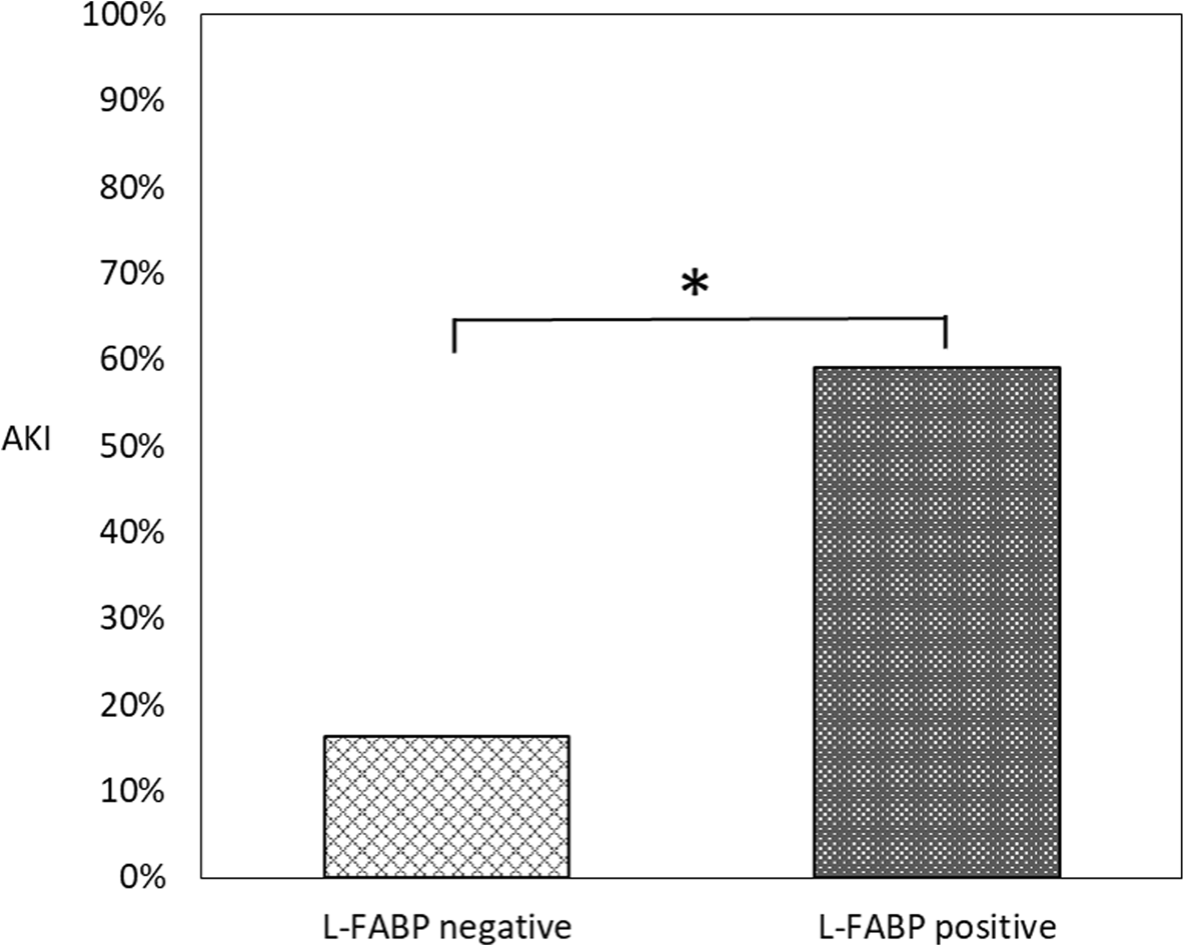
In the two groups, after exclusion patients already progressed to AKI at admission, urinary liver-type fatty acid-binding protein (L-FABP) negative and positive and the proportion of acute kidney injury (AKI) are shown. *p < .05 using Fisher’s exact test

After exclusion, 86 in negative group and 22 in positive patients remained. (Table 2) Only the rate of persistent AKI showed significant difference. In both groups, there was no significant difference in serum creatinine. KDIGO class showed significant difference. And the rate of onset of AKI is higher in the positive group. The rate of AKI is 16.3% in the negative group and 59.1% in the positive group, respectively. And there was a significant difference between the two groups (*p* < 0.001) (Fig. 2).

**Table 2:**
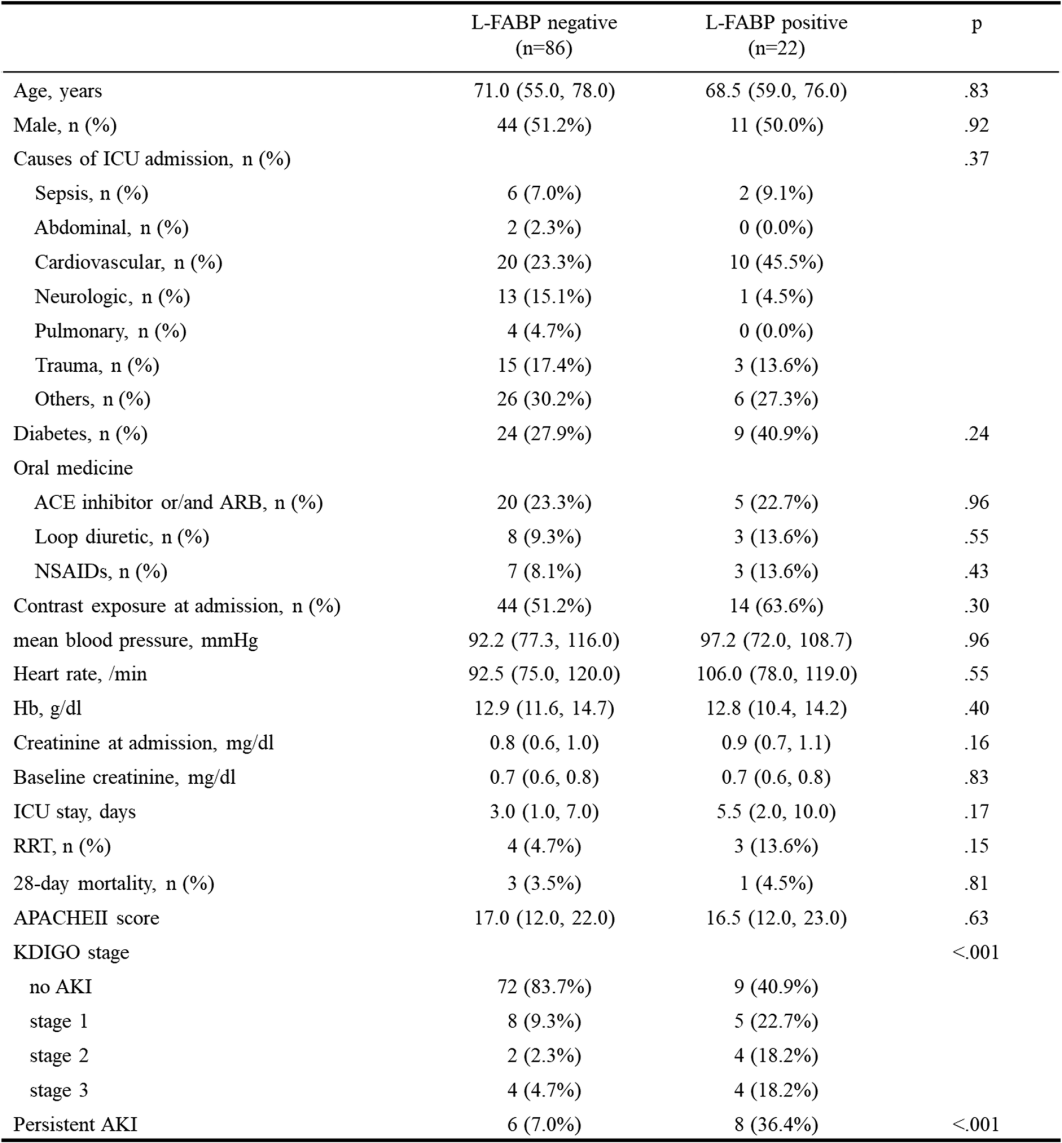
Characteristics of the patients not diagnosed as AKI from creatinine at admission

*L-FABP* liver-type fatty acid-binding protein, *ICU* intensive care unit, *ACE* angiotensin-converting enzyme, *ARB* angiotensin II receptor blocker, *NSAIDs* non-steroidal anti-inflammatory drugs, *RRT* renal replacement therapy, *APACHE* Acute Physiology and Chronic Health Evaluation, *KDIGO* Kidney Disease: Improving Global Outcomes, *AKI* acute kidney injury

## Discussion

### The strong points of this study

L-FABP is a low molecular weight protein that is expressed in the cytoplasm of human proximal renal tubule epithelial cells. Fatty acids in cells are transported in subcellular organelles to maintain the homeostasis of fatty acids. In addition, it reflects renal tubular disorder and is said to be excreted through urine [5]. Urinary L-FABP has been reported to enable early prediction of the onset of AKI with the conventional diagnostic criteria after open heart surgery and in sepsis [5–8]. Moreover, a study also reported that the onset and prognosis of AKI could be obtained in a mixed ICU [12]. However, no studies summarizing urinary L-FABP concentrations in various types of critically ill patients who are transported to emergency and critical care centers have been conducted. To the best of our knowledge, this is the first study to determine whether the onset of AKI can be predicted from urinary L-FABP concentration in the emergency field. Though few studies used the rapid semiquantitative assay kit, according to the report by Sato et al. [13], the results of rapid assay kits on urinary L-FABP concentration well correlate with quantitative results and are also superior in predicting the onset of AKI. The simplicity of the measurement process and the rapid speed at which the results can be obtained are also characteristics of this study. In quantitative tests, at least a few days are required for the results on urinary L-FABP concentration to be obtained. Even when there are variations in the background and type of disease, the fact that onset of AKI can be predicted from the results obtained at that one time when the sample is collected in the initial treatment and, moreover, in about 15 min when the rapid kit is used is also beneficial clinically. Among patients that are transported to emergency centers, there are often those whose condition worsens within minutes or tens of minutes. Thus, the introduction of the rapid RRT by which urinary L-FABP concentration can be measured within 15 min is very useful.

### Predicting AKI

In this study, the rapid assay kit has good specificity. The kit can find patients already progressed to AKI and, even if one’s creatinine level was not increased yet, predict the onset of AKI after admission. However, the negative group had many false negatives. With regard to the sensitivity of the rapid assay kit, Sato et al. also mentioned that an improvement is desirable, and that will be a future topic. Although the results of the urinary L-FABP concentration are not the criteria for introducing RRT, based on the results of this study, we may be able to find the criteria with using L-FABP data.

### Persistent AKI

Recently, the concept of “persistent AKI” was proposed for AKIs predicted to persist for at least 3 days and for which a positive intervention would be most likely required [14]. Matsuura et al. also defined persistent AKI as stage 2 or 3 AKI [15]. We used latter definition in this study because there were patients who were discharged within 72 h. Moreover, the renal angina index [16, 17] is used to predict AKI. Furthermore, it has also been reported that inclusion of urinary L-FABP results increases the predictability of persistent AKI [15]. These are studies with an impact; however, they are all studies involving the ICU. Thus, when quick judgment needs to be performed in urgent cases, the rapid assay kit such as that used in this study will be useful for predicting serious AKI from the vital signs of the patients upon their arrival at the hospital. In this study, the assay kit can predict persistent AKI at admission even if patients’ creatinine levels were not increased yet.

### Limitations

This study has several limitations. First, it is a single-center study, and the sample size is small. Therefore, a large-scale multicenter study is necessary. Next, a semiquantitative rapid assay kit was used, and therefore, the judgment becomes somewhat subjective. Moreover, L-FABP concentrations for introducing RRT cannot be decided. In the future, we would like to plan a quantitative investigation.

## Conclusion

The onset of AKI can be predicted from urinary L-FABP concentrations determined using a rapid assay kit in patients transported to the emergency and critical care center after their admission.

## Data Availability

The datasets used and analyzed during the current study are available from the corresponding author on reasonable request.

## Abbreviations

ACE: Angiotensin-converting enzyme
AKI: Acute kidney injury
APACHE: Acute Physiology and Chronic Health Evaluation
ARB: Angiotensin II receptor blocker
DNAR: Do not attempt resuscitation
GFR: Glomerular filtration rate
ICU: Intensive care unit
KDIGO: Kidney Disease: Improving Global Outcome
L-FABP: Liver-type fatty acid-binding protein
NSAIDs: Non-steroidal anti-inflammatory drugs
RRT: Renal replacement therapy
SOFA: Sequential organ failure assessment
